# Conservative surgical management of simple monostotic fibrous dysplasia of the proximal femur in a 19-year-old basketballer: a case report

**DOI:** 10.1186/s13256-018-1763-3

**Published:** 2018-08-31

**Authors:** David Yung, Kazutaka Kikuta, Tetsuya Sekita, Naofumi Asano, Robert Nakayama, Masaya Nakamura, Morio Matsumoto

**Affiliations:** 1 Department of Orthopaedic Surgery, Keio University School of Medicine, 35 Shinanomachi Shinjuku-ku, Tokyo, 160-8582 Japan; 2 Royal College of Surgeons in Ireland, 123 St Stephen’s Green, Dublin 2, Ireland

**Keywords:** Fibrous dysplasia, Femoral neck, Fibula strut, Autologous bone graft, Implants

## Abstract

**Background:**

Fibrous dysplasia is a rare benign, intramedullary, fibro-osseous lesion. It is thought to be a developmental disorder of bone maturation where normal lamellar bone is replaced by irregular trabecular bone ensnared with fibrous dysplastic tissue that is unable to complete maturation resulting in significant loss of mechanical strength. This, together with the inability to mineralize sufficiently, leads to deformity, pain, and pathological fractures. It typically presents in young adults, with an equal representation in both genders. Surgical intervention is necessary in mild cases with chronic symptoms to prevent pathological fractures or to correct deformities.

**Case presentation:**

A 19-year-old Chinese woman presented with non-traumatic, nonspecific left hip pain during basketball training. X-rays demonstrated a ground glass lesion, 10 cm in length, in her left femoral neck, which is a classic sign of fibrous dysplasia. No other deformities were noted. She was managed conservatively with analgesia for 6 months; however, her condition did not improve and a decision was made for surgical intervention. The lesion was a type 1 lesion according to the Ippolito radiological classification of fibrous dysplasia, which is a lesion with mild deformities. Therefore, we performed minimal curettage and insertion of a free autologous fibula strut harvested from her left leg, for structural stability. No implants were used. The operation was successful and her postoperative course was uneventful. Histology confirmed the diagnosis of fibrous dysplasia. She achieved partial weight bearing at 4 weeks postoperation, and full weight bearing at 8 weeks, and returned to basketball at 12 weeks. At 1-year follow-up, she returned to competitive basketball and remained pain free with no complications.

**Conclusions:**

Fibrous dysplasia is a rare and benign fibrous tumor of the bone that presents mostly in a young patient population. From our case, we have shown that it is possible to treat young patients with uncomplicated Ippolito type 1 fibrous dysplasia with a minimally invasive approach of using a cortical bone graft for structural augmentation of the affected area, without the use of implants. They are able to fully return to an active and vigorous lifestyle without restriction of activities or long-term risks of orthopedic implant complications.

## Background

Fibrous dysplasia (FD) is rare benign bone tumor; surgical management of FD remains difficult in young patients. FD is an intramedullary, fibro-osseous lesion that was first described by Lichtenstein and Jaffe [1] in 1942. The prevalence of FD is estimated to be 5–7% of all bone tumors [2]. It is thought to be a developmental disorder of bone maturation where normal lamellar bone is replaced by irregular trabecular bone ensnared with fibrous dysplastic tissue that is unable to complete maturation resulting in significant loss of mechanical strength. This, together with the inability to mineralize sufficiently, leads to deformity, pain, and pathological fractures. It mostly affects children and young adults of both sexes, with the majority of cases presenting by the third decade of life.

FD has been linked to missense mutations in the guanine nucleotide-binding protein/α-subunit (*GNAS*) gene (20q13), resulting in the dysplastic features [3]. These mutations have been found to be present in 93% of cases [4]. FD can either be monostotic or polyostotic, with the monostotic form being six times more prevalent. Young patients with monostotic FD often present hip pain or fatigue fractures, while those with the polyostotic form often develop significant limb deformities by adolescence [4, 5]. Malignant mutation is rare, and the majority of cases are mild and can be managed conservatively. However, surgical intervention is required for mild cases with problematic chronic symptoms to prevent pathological fractures or for the correction of deformities [3, 5]. At present, there is no definitive surgical strategy for these mild cases of FD.

## Case presentation

**Fig. 1:**
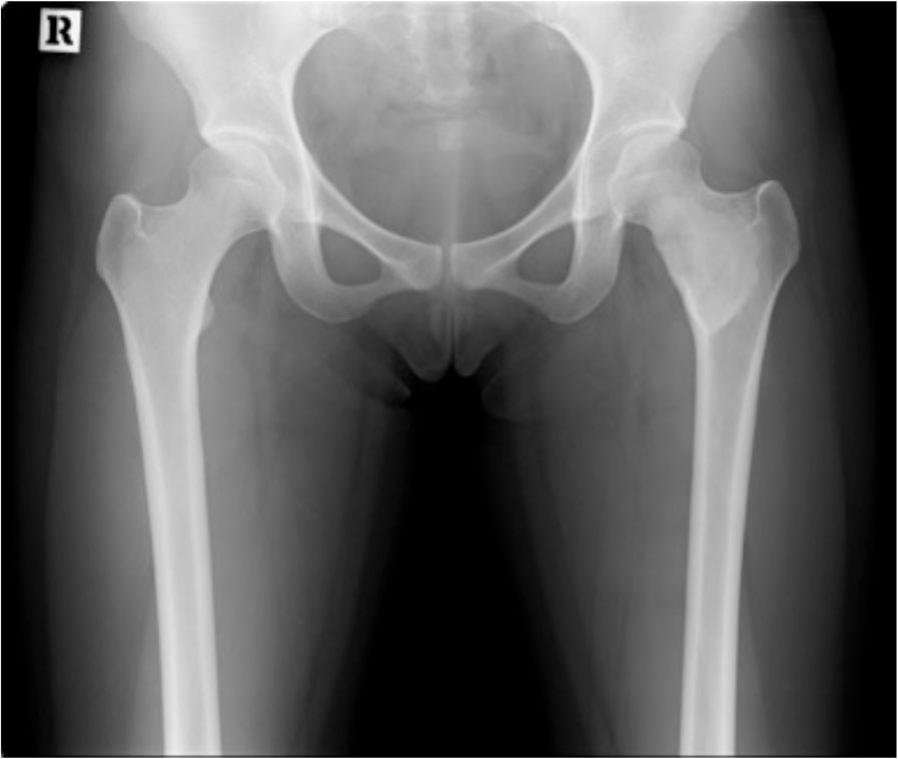
X-ray of the hip, demonstrating ground glass lesion in left neck of femur

**Fig. 2:**
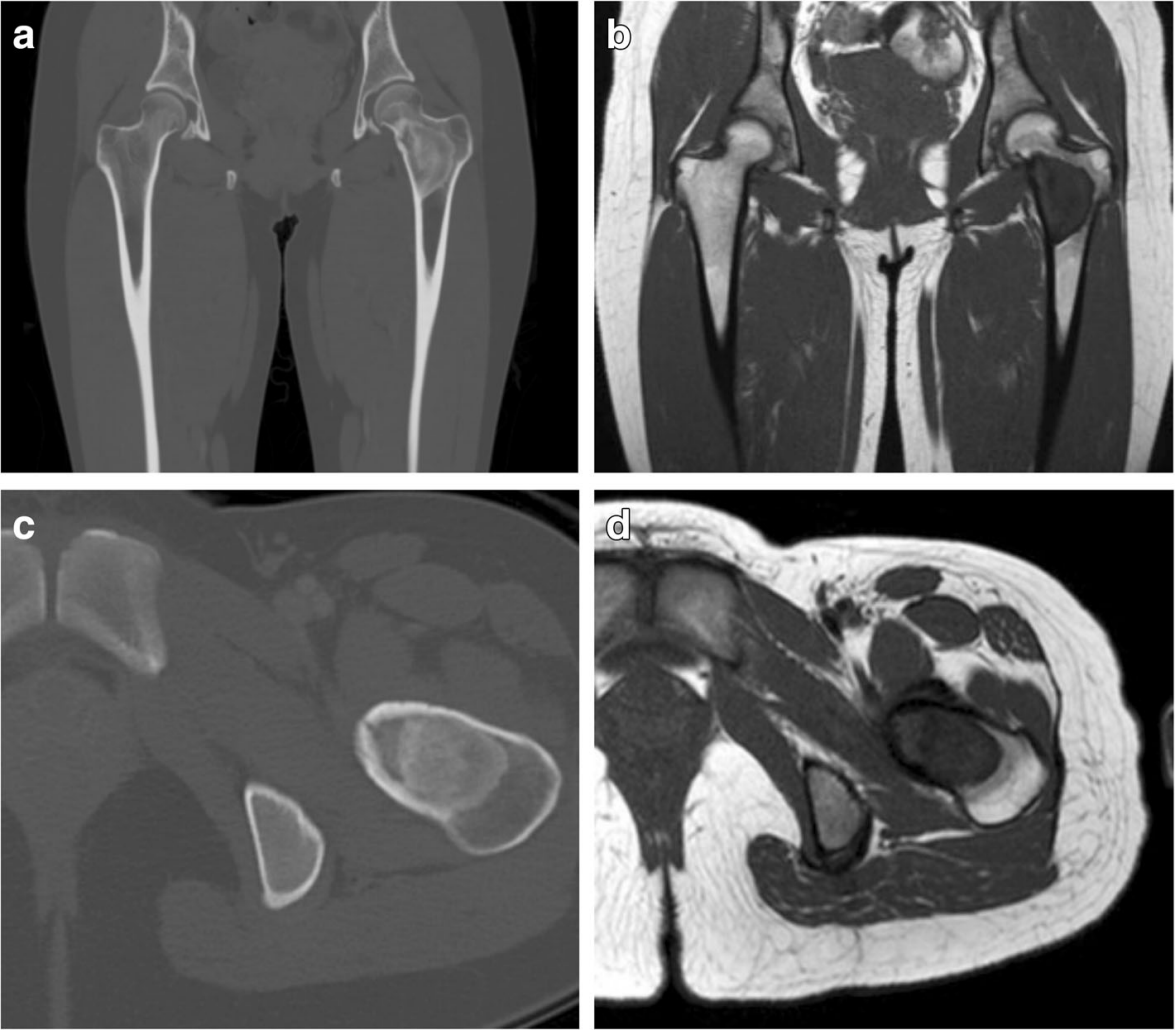
Preoperative CT and magnetic resonance images of proximal femoral lesion. **a** and **b** CT, coronal and axial views of proximal femur. **c** and **d** T1-weighted magnetic resonance images, coronal and axial views of proximal femur

A 19-year-old Chinese woman was admitted to our institution for the treatment of monostotic FD of her femoral neck. She presented in June 2015 with non-traumatic, nonspecific left hip pain that occurred during university basketball training. She first noticed the pain a month before and visited an out-patient clinic in another hospital when the pain did not improve. She denied any injury to the area or loss of range of motion (ROM), and her condition was not improved by analgesia. X-rays of the region demonstrated a fairly extensive ground glass lesion (Fig. 1) in her left femoral neck, which is a classic sign of FD. Computed tomography (CT) and magnetic resonance imaging (MRI) revealed the lesion to be 10 cm in length (Fig. 2). She was managed conservatively for 6 months; however, because the pain did not improve she was referred to Keio University Hospital for management. She opted for surgical treatment of the lesion for pain relief and to prevent future fractures and to return to her active lifestyle. She has no other significant past medical history.

**Table 1:** Surgical approach to augmentation of fibrous dysplastic head of femur with autologous fibula bone graft

1. Prior to surgery, the length of fibula graft required is measured according to radiographic measurement of the length of the patient’s femoral neck to the femoral head. The graft is then harvested and care is taken to preserve the periosteum.	
2. The defect created is then filled with cylindrical artificial bone and closed.	
3. The patient is then placed in a traction bed in the lateral position, similar to neck of femur fracture surgery.	
4. The femoral head is first deployed by the lateral approach.	
5. A drill is then used to create space for the graft in the same manner as inserting a lag screw.	
6. The fibular is then inserted into the created hole and hammered into place.	
7. A drain is inserted and the wound closed in layers.

**Fig. 3:**
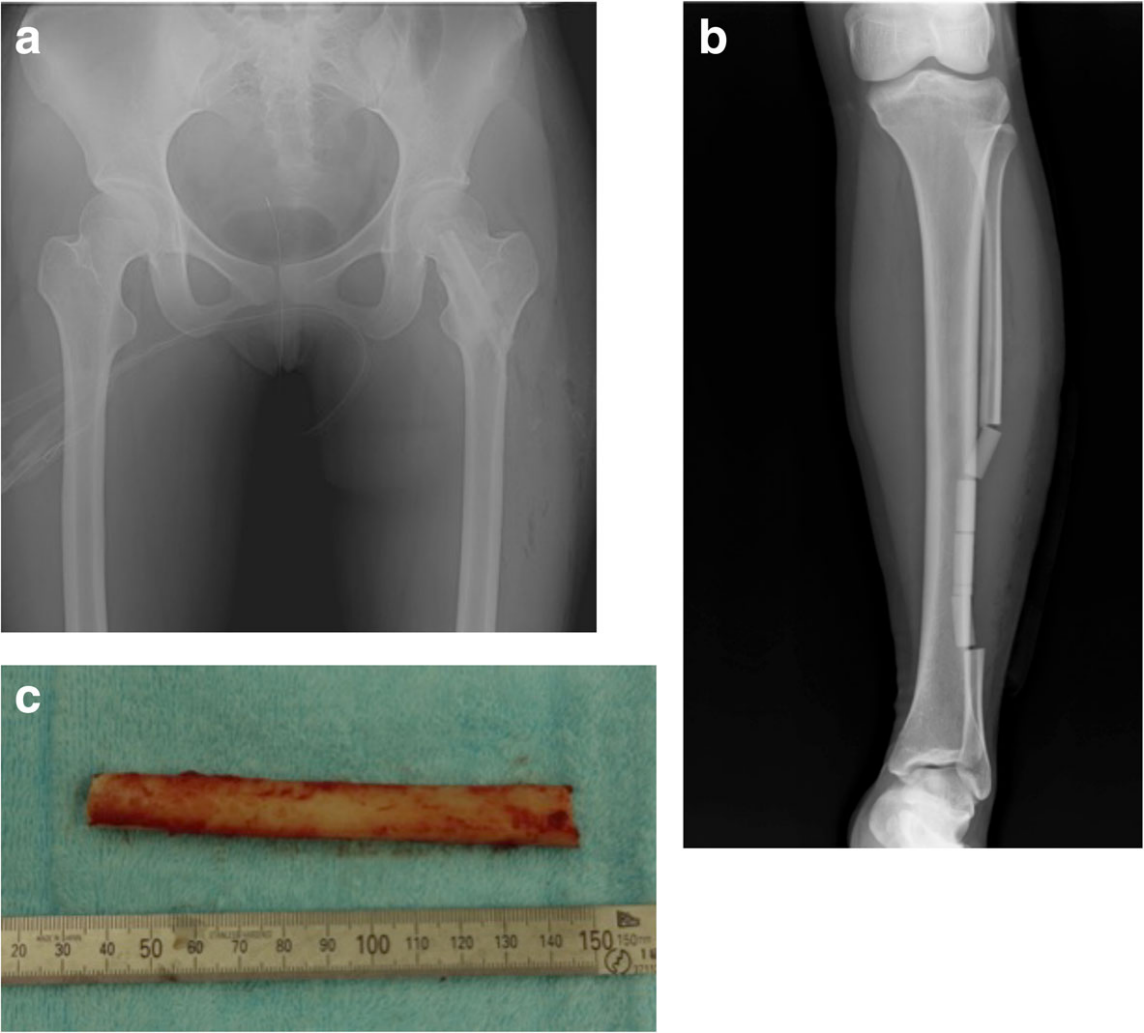
Postoperative radiograph of the neck of femur with left autologous fibula graft strut inserted. **a** Postoperative radiograph of the neck of femur. **b** The defect in the fibula was filled with β-tricalcium phosphate (Superpore®; HOYA Technosurgical Inc., Japan). **c** left autologous fibula graft strut inserted

In July 2016, she underwent surgery to augment her left femoral neck with an autologous cortical, free fibula bone graft. A minimal curettage and lavage of the femoral neck was done to remove fibrous dysplastic tissue to make room for the graft. The free fibula graft was then harvested from her left fibula. The graft was 10 cm long and hammered into the femoral defect to act as an intramedullary pin. No screws or plates were used and the defect in the fibula was filled with β-tricalcium phosphate (Superpore®; HOYA Technosurgical Inc., Japan) (Fig. 3 and Table 1). Samples of the tissue were sent to our pathology department for histological analysis, which confirmed the diagnosis of FD. There were no intraoperative complications and her postoperative course was uncomplicated; a short course of prophylactic cefazolin and adequate analgesia were prescribed. She discharged with non-weight bearing (NWB) on crutches on postoperative day 7.

**Fig. 4:**
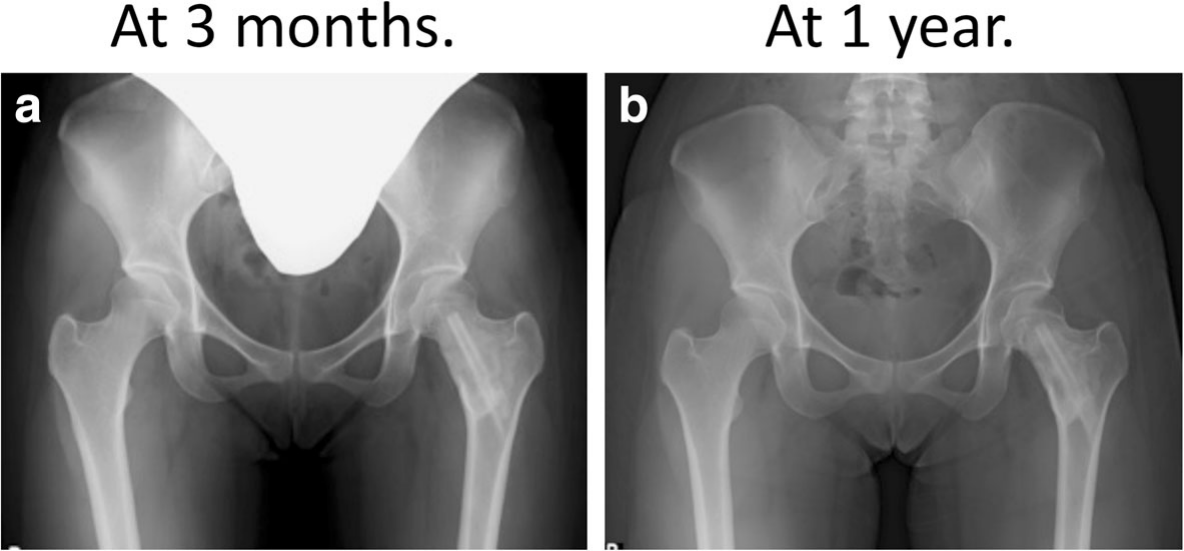
The patient’s neck of femur. **a** 3-month follow-up; **b** 1-year follow-up

As no dynamic hip screw was used, she was NWB for 6 weeks to allow for adequate healing and integration of the bone graft. With physiotherapy, she returned to partial weight bearing (PWB) at 4 weeks postoperation, full weight bearing (FWB) at 8 weeks, and returned to her sporting activity at 3 months. The operative site and the donor site have healed well and her postoperative course has been unremarkable. Follow-up X-rays demonstrated healing and integration of the femoral graft without reabsorption and with no recurrence of FD (Fig. 4). She was followed up regularly for a year to monitor for reabsorption of the fibula graft, which is a common complication of autologous bone grafting. At 1-year follow-up, she had completely returned to competitive basketball, recently taken up tai-chi, and had no limitation of activities and no residual hip pain.

## Discussion

FD is a benign bone tumor. Most cases present as monostotic lesions, with a peak incidence in young patients in the second decade of life. Malignant transformation is also a very rare occurrence in FD [6]. The proximal femur is a common site of FD and results in microfractures that cause pain, deformity, and the loss of biomechanical strength and stability. Most of these lesions, however, present with very mild deformities in the type 1 pattern described in the radiological classification of proximal femoral FD by Ippolito *et al.* [7]. This subgroup maintains the biomechanical stability of the femoral head, without deformity or loss of strength, and rarely progresses to the more severe forms; this makes them good candidates for conservative surgical intervention for pain relief, prevention of pathological fractures, as well as structural augmentation to prevent further deformities from occurring.

Surgical management in these cases should therefore be as minimally invasive as possible, with the aim for the patient to make a complete return to the rigorous daily activities and hobbies of youth as soon as possible, as well as to prevent pathological fractures. However, a definitive surgical strategy has yet to be established. Hence, in this case, we performed only a cortical strut autograft of fibular bone without a full tumor curettage or internal fixation in order to prevent future pain, microfractures, and deformities of the femoral neck. Due to the benign nonaggressive nature of the tumor [8] and the extremely low risk of malignant transformation, a complete curettage of the lesion is unnecessary in type 1 deformities [3, 7]. Further, the intent of this report is to demonstrate that only augmentation with an autologous fibula strut is necessary in managing younger patients with FD and prevents any of the risks and complications of instrumentation.

Bone grafts are the ideal filler agent following curettage of the proximal femur. They are osteoinductive, osteoconductive, and osteogenic, which facilitates the organic incorporation of the graft at the transplant site [5, 9, 10]. Bone grafts also achieve sufficient biomechanical strength in the long term. Non-vascularized cortical bone grafts are the choice graft for transplantation in proximal femoral FD. They provide excellent initial structural support, while retaining some osteoinductive, osteoconductive, and osteogenic properties [11]. In a report by Enneking and Gearen on 15 patients with mild deformities treated with autologous cortical bone grafts, radiographs continued to demonstrate continuity and integrity of the grafts in all 15 patients at final follow-up [12]. Of those patients, ten also demonstrated significant re-ossification within the transplant site and a decrease in the size of the lesion [12]. Cancellous bone grafts are no longer used in FD treatment due to the high reabsorption rate and replacement of the graft by dysplastic tissue, which results in recurrence of the disease [3, 5, 9]. Further, cancellous grafts do not provide any substantial structural support initially, and only achieve strength after 6–12 months [11].

Autologous bone grafting has been the traditional approach in the treatment of FD, although, recently, allogenous grafts have become more popular [3, 5, 9]. Autologous grafts are generally accepted as the gold standard of bone grafting and have the advantage of faster incorporation, with lower risk of infection at the transplant site. However, there are disadvantages to using autologous grafts including limited supply, requiring a second surgical site for harvesting the graft, donor site weakening, and increased postoperative morbidity [3]. Therefore, several authors have argued for the advantages of allogeneic bone grafts over autologous bone grafts in FD treatment [3, 5]. These advantages are the slower rate of reabsorption, the elimination of donor site morbidity, and the great availability of the grafts. However, graft availability depends on the existence of a bone bank, which is not available in Japan due to cultural reasons. Further, allogenic grafts do not have osteoinductive or osteogenic properties and, thus, are associated with a slower rate of incorporation. They also carry a theoretical risk of infectious disease transmission [11]. Hence, autologous bone grafts provide a better option for bone grafting.

Previous reports have researched the various surgical strategies revolving around curettage of the lesion followed by bone grafting with the option of additional strengthening by internal fixators [3, 5]. Most reports of the surgical treatment of FD do not involve the use of internal fixation following the bone graft. A canine study showed that in non-vascularized cortical grafts, there is a transient loss of mechanical strength at about 6 weeks due to the remodeling process; however, strength is regained within a year [11]. Hence, without internal fixators, there is in theory a risk of fracture of the operated site within this period. Thus, in the study by Enneking and Gearen, patients were NWB on crutches for 6 weeks, followed by PWB and gradual return to FWB [12]. However, this is a rather long period to restrict a young patient from their activities. Hence, DiCaprio and Enneking, in their review, recommend internal fixation for large lesions [5]. A recent study by Nishida *et al*. on eight patients with mild FD of the proximal femur also explored the use of internal fixation with dynamic hip screws in speeding up the return to FWB status and thus to regular activities [3]. They reported that five of their eight patients were able to achieve FWB by 2 weeks after surgery [3].

The use of internal fixation in younger patients is not without complications. First, in pediatric patients with open physes, there is a risk of causing physeal arrest and closure through penetration of the epiphysis by the implant [13–15], which can result in a limb length discrepancy. Because the proximal physis contributes only 15% of bone length and tends to close first, this discrepancy may not be clinically significant in older children and adolescents, but it can be significant in younger children [16]. Second, although it is possible to return to an active lifestyle, even to playing contact sports at a professional level [17], there are long-term risks associated with the retention of implants in young patients, which include the risk of peri-implant fractures, implant migration and penetration, implant failure, and infection [15, 18]. As a result, many hospitals and centers advocate the routine removal of implants at some point after bone union and healing have occurred, especially in younger patients [15, 18]. Third, FD also has a greater rate of recurrence in children, whose bone is still immature [5]. In the event of a local recurrence, previous instrumentation complicates future surgeries due to implant embedment, as well as local fibrosis and scarring [15]. In our experience, there is also a risk of recurrence occurring along the implant tract. Therefore, it seems unnecessary to place young patients with stable Ippolito type 1 under these risks as they do not have extensive lesions causing significant structural stability, pathological fractures, or deformities that require correction.

Without instrumentation, our patient was able to achieve FWB status within 8 weeks, which is similar to the results reported by George *et al.*, whose patients with FD achieved FWB between 6 and 14 weeks [10]. Two of those patients, who were similar in age and symptomology to our patient, were managed to achieve FWB within 6 and 8 weeks [10]. Further, while George *et al*. restricted their patients from fully resuming all activities until 12 months after their procedure, which is a rather long time to prevent a young patient from returning to their active lifestyle, our patient was able to return to sports at 12 weeks after her operation uneventfully. At 1-year follow-up, she had fully returned to competitive basketball, as well as taken up tai chi, and is unrestricted in her activities. A 1-year follow-up period is necessary, despite returning to sports at 12 weeks, because it is important to monitor the patient for reabsorption of the autologous bone graft. Overall, this suggests that treating patients with Ippolito type 1 FD with only an autologous bone graft without internal fixation does not result in mechanical instabilities and does not require a long recovery time.

## Conclusions

Our case has demonstrated that it may be possible to treat simple cases of Ippolito type 1 FD only with fibula strut augmentation without instrumentation, whereas cases of more complicated FD that involve extensive lesions, deformities, or pathological fractures may warrant internal fixation for mechanical stability and correction of deformity. In simple cases involving young patients, the complications associated with implants can be avoided safely with a conservative surgical approach, and an earlier return to an active and vigorous lifestyle can be achieved.

## Abbreviations

CT: Computed tomography
FD: Fibrous dysplasia
FWB: Full weight bearing
*GNAS*: Guanine nucleotide-binding protein/α-subunit
MRI: Magnetic resonance imaging
NWB: Non-weight bearing
PWB: Partial weight bearing
ROM: Range of motion
